# Rediscovering local breeds of naturally free-range hens: a survey on Italian consumers’ awareness of hen welfare and egg purchasing behavior

**DOI:** 10.1186/s12917-025-04971-x

**Published:** 2025-10-21

**Authors:** Elena Gosso, Greta Falavigna, Valentina Lamonica, Cristina Lamberti, Simona Cirrincione, Laura Cavallarin, Maria Gabriella Giuffrida, Achille Schiavone

**Affiliations:** 1 University of Gastronomic Sciences, Piazza Vittorio Emanuele 9, Pollenzo, CN 12042 Italy; 2 Department of Veterinary Sciences, University of Turin, Largo Paolo Braccini 2, Grugliasco, TO 10095 Italy; 3 Research Institute on Sustainable Economic Growth (IRCrES), CNR, Strada delle Cacce 73, Turin, 10135 Italy; 4 Institute of Science of Food Production (ISPA), CNR, Largo Paolo Braccini 2, Grugliasco, TO 10095 Italy

**Keywords:** Hen egg, Hen welfare, Animal welfare, Consumers, Italian local hen breeds, Willingness to pay, Artificial neural network, Garson indexes

## Abstract

**Background:**

Egg production is dominated by the use of conventional cage systems, but the increasing awareness of consumers about animal welfare has led to a shift toward cage-free and free-range systems. Italian consumers are increasingly valuing farming methods and hen welfare in their purchasing decisions. Moreover, native poultry breeds are gaining attention for environmental sustainability reasons and their contribution to biodiversity. This study explores Italian consumers’ awareness, attitudes, and knowledge about eggs from local hen breeds raised in high-welfare, free-range systems. To this end, a survey was proposed, through a Computer Assisted Web Interviewing (CAWI) approach, and administered to egg consumers *via* social media.

**Results:**

The survey collected 1,222 responses, mainly from women and individuals under 75. Two key questions assessed the consumers’ attention toward “animal welfare” and “hen welfare”. The results of descriptive statistics showed that 27.5% of the respondents were attentive to animal welfare, while 61.2% frequently or always considered how hens were raised when they bought eggs. The main result of econometric analyses on the “animal welfare” model suggested that the *welfare-quality* variable was positively, statistically significant with the highest odd-ratio. The *size of the family* and *eating meat* were instead negatively, statistically significant. Similar results were found for the “hen welfare” model, even if the studying or working in an agri-food sector outcomes were not significant. A stepwise logistic regression and Artificial Intelligence-based analysis proved to be highly effective in identifying and prioritizing the characteristics that defined the consumers’ awareness of local hen breeds. Welfare-quality was the most important feature for these consumers when purchasing eggs. Egg consumers are in fact willing to pay more for eggs from well-raised and/or local breed hens.

**Conclusion:**

The study has shown there is growing attention to animal and hen welfare and egg production methods. These results highlight a cultural dimension of hen welfare and hen breed awareness, thus suggesting that policymakers should promote the education of consumers on animal farming practices and local hen breeds, thereby achieving protection of their genetic heritage.

**Supplementary Information:**

The online version contains supplementary material available at 10.1186/s12917-025-04971-x.

## Background

Eggs are an important and cheap source of nutrients, including high quality proteins a well-balanced amino acid composition, essential polyunsaturated fatty acids (PUFA), carotenoids, and vitamins. They are consumed worldwide, accepted by almost all the consumers and they are mainly not under religious diet restrictions [1].

The global production of eggs has increased by 40% over the past decade and reached 97 million tonnes in 2023. Asia is the leading producer of fresh hen eggs, with 46 million tonnes per year, and it accounts for 64% of the global production, followed by the Americas (20%), Europe (12%), Africa (4%) and Oceania (0.4%) [2]. Domestic production in Italy, after a slight decline in 2022, likely due to the Russia-Ukraine conflict and to avian flu (−0.6% in 2021), recovered in 2023 (+ 3.5%) and further increased in 2024 (+ 4.5%) [3, 4]. This increase may be attributed to the low price of eggs, compared to other animal protein sources, and to the eradication of their historical link to cardiovascular disease [5]. Indeed, whole eggs are a source of dietary cholesterol (185–200 mg/egg), and the popular belief is that egg consumption can influence blood cholesterol levels [6]. However, in 2018, Kim and Campbell reported the results of two randomized-crossover studies that demonstrated that one egg per day did not increase the total cholesterol concentration in the plasma, because the cholesterol contained in whole eggs is not well absorbed [7]. Drouin-Chartier and colleagues reported similar findings in 2020 in a systematic review and meta-analysis of prospective cohort studies involving 173,590 women and 42,055 men, all of whom were free of cardiovascular disease [8]. Egg consumption is in fact associated with a lower risk of hypertension (a major cause of cardiovascular diseases in an aging population), whereas red meat and poultry consumption is linked to a higher risk [9]. An Italian study, conducted on an older population living in the Southern Mediterranean area, demonstrated a protective role of eggs against both hypertension on its own and when associated with steatotic liver disease. The study showed that the risk of developing these pathological conditions decreased linearly as egg consumption increased (> 3 eggs per week) [10]. Egg consumption may also have a positive effect on the prevention of seniority diseases, such as osteoporosis, as eggs are an important source of vitamin D3 and 25-hydroxyvitamin D_3_(25(OH)D_3_) (which is important because it is adsorbed quickly), and Alzheimer’s disease, thanks to the high levels of choline, DocosaHexaenoic Acid (DHA), and tryptophan in eggs [11, 12].

The production of eggs began to increase significantly throughout the world in the 20th century, and especially from the mid-1950s onwards. A key factor in this growth was the use of conventional cage breeding for laying hens. Conventional cages generally have a surface of 550 cm^2^/hen, and, although they were banned in Europe in 2012, they are still in use in other parts of the world, such as the U.S.A., Mexico, Brazil, China, and Japan. In Europe, the farming methods for laying hens are regulated within the framework of the directive on the welfare of laying hens [13]. Indeed, only the so-called “enriched cages”, with a surface of 750 cm^2^/hen (600 cm^2^ “usable” area), are allowed, so maximum 13 hens/m^2^. Other alternative systems that avoid cage use are also allowed: “barns” without outdoor access and “free-range” systems with outdoor access, both of which should not exceed 9 hens/m^2^ “usable” area. Moreover, the “organic” option was introduced by EU regulation 2018/848 [14], and subsequently implemented by EU regulation 2020/46 [15], whereby 6 hens/m^2^ of “usable” area was regulated, with an outdoor area of 4m^2^/hen usable for a minimum of 1/3 of their life. Moreover, routine beak trimming was prohibited by this regulation, although it was permitted for other farming methods [16]. According to all the laying hen production systems, in 2004, the EU (including the UK) introduced mandatory codes for eggshells [17]. These codes specify, apart from the origin of the eggs, the method of production: the number 3 is indicated for “enriched cage”, 2 for “barn”, 1 for “free-range” and 0 for “organic”. European data on laying hens reared in 2022 showed that: 39.7% were kept in enriched cages (code 3), 37.8% in barns (code 2), 15.5% in free-range systems (code 1), and 7.1% were reared in organic systems (code 0) [18]. The most common rearing method in Italy is “on the ground,” (barn, code 2), and it accounts for 54% of the total livestock. Only 36% of the hens in Italy are raised in enriched cages (code 3), and 10% are kept outdoors (4,9% free-range code 1 and 5% organic code 0) [3].

However, consumers’ attitudes, driven by changes in the European regulations and an increased awareness of animal welfare, are changing. Several surveys conducted in different countries have investigated consumers’ perceptions of laying hen [19–23].

The general consensus is that the public perceives free-range systems more positively, in terms of health, stress levels, behavior, and the environmental impact of hens, than conventional caged systems [19]. However, the price of eggs significantly influences purchasing decisions in some countries, such as Croatia [24]. An online survey was administered to nine countries in Europe (Finland, the United Kingdom, France, Italy, Belgium, Germany, the Netherlands, Romania, and Denmark) to establish the consumers’ views on a responsible egg production [18]. In general, the consumers involved in the study considered sustainability attributes to be important in assessing egg product quality. In Germany, a responsible production was valued more highly than product qualities, while in the Netherlands and Romania, product attributes were prioritized over responsible production. However, despite many research articles having been published, the impact of alternative production systems on egg quality remains controversial, although these systems should satisfy the ethical needs of the consumers [1]. Italy, like France and the UK, places significant importance on local/regional origin and on the farming methods (free-range and organic) in consumers’ purchasing decisions, even though organic farming systems in Italy account for only 4.9% of the total egg production [18].

Italian consumers are also showing increasing interest in ancestral food systems. Although modern egg production relies on hybrid laying hens, selected because of their high performance, certain advantages in adopting alternative systems, based on local breeds reared extensively under free-range conditions, are recognized. In Italy, 53 native chicken breeds have been described to date, the majority of which are reported to be endangered or extinct. Fortunately, the last few decades have witnessed significant growing interest in native breeds, and 22 breeds are currently included in the National Registry of Autochthonous Poultry Breeds, established in 2014 by the Italian Ministry of Agriculture [25].

These breeds are distributed across Italian regions, each with unique morphological and productive traits. For example, in Piedmont, a region in the Northwest of Italy, three local hen breeds have been rediscovered and preserved: Bionda Piemontese, Bianca di Saluzzo, and Millefiori Piemontese. These breeds, such as the Livorno breed, which was the first to be officially registered, are part of Italy’s regional heritage and demonstrate a better adaptability to emerging challenges, such as climate change [26]. Although local breeds may offer a reduced egg production, they are appreciated because of their adaptability to diets with poor nutrients and bad digestibility, and for their better resistance against infectious diseases. Thus, the promotion of this type of production is more suitable for local distribution, thereby helping to mitigate environmental impacts. Their promotion thus ensures greater attention to biodiversity and represents an effort in the direction of sustainable productivity [27]. A recent Italian survey on consumers’ perception of the product quality of eggs from native poultry breeds revealed a preference for meat and eggs from local hen breeds (LHB), which were associated with superior organoleptic properties and higher nutritional quality than products from commercial lines [28].

The aim of this study has been to obtain some preliminary insights into consumers’ attitudes, preferences and possible willingness to pay for eggs produced from LHB and raised in free-range systems with high animal welfare standards. To this end, a survey was designed to analyze the Italian consumers’ attitudes towards preserving general animal welfare, with a specific focus on hen welfare and consumers’ knowledge about LHB, which represent the novelty of this study. The survey was proposed through a CAWI (Computer-Assisted Web Interviewing) approach and administered to egg consumers *via* social media. The CAWI methodology is widely used in scientific research because of its ease of administration. It involves designing an online questionnaire and distributing it through the channels best suited to the survey objectives. Most national and international surveys are conducted, at least in part, using this method, as it allows researchers to efficiently reach the target population. This approach has gained widespread acceptance in the scientific community, particularly within qualitative research, where it is increasingly adopted as a reliable tool for gathering empirical data [29, 30].

## Results

A total of 1,222 answers to the distributed survey were collected. There was a prevalence of female respondents (843 women, 374 men, and 5 Non-binary/Prefer not to disclose), and most of the respondents were under 75 years of age (about 1% over 75 years of age). Details about the full survey used in this study and its results are reported in Supplementary File 1_Survey and Supplementary File 2_Data Collection, respectively.

The descriptive statistics suggested that 27.5% of the consumers paid attention to animal welfare (i.e., they responded “I am very attentive to it”), while 5% was not interested in animal welfare or was indifferent to it. In this case, the descriptive statistics showed that most of the consumers paid attention to hen welfare (about 61% answered “Often” or “Always”).

**Fig. 1:**
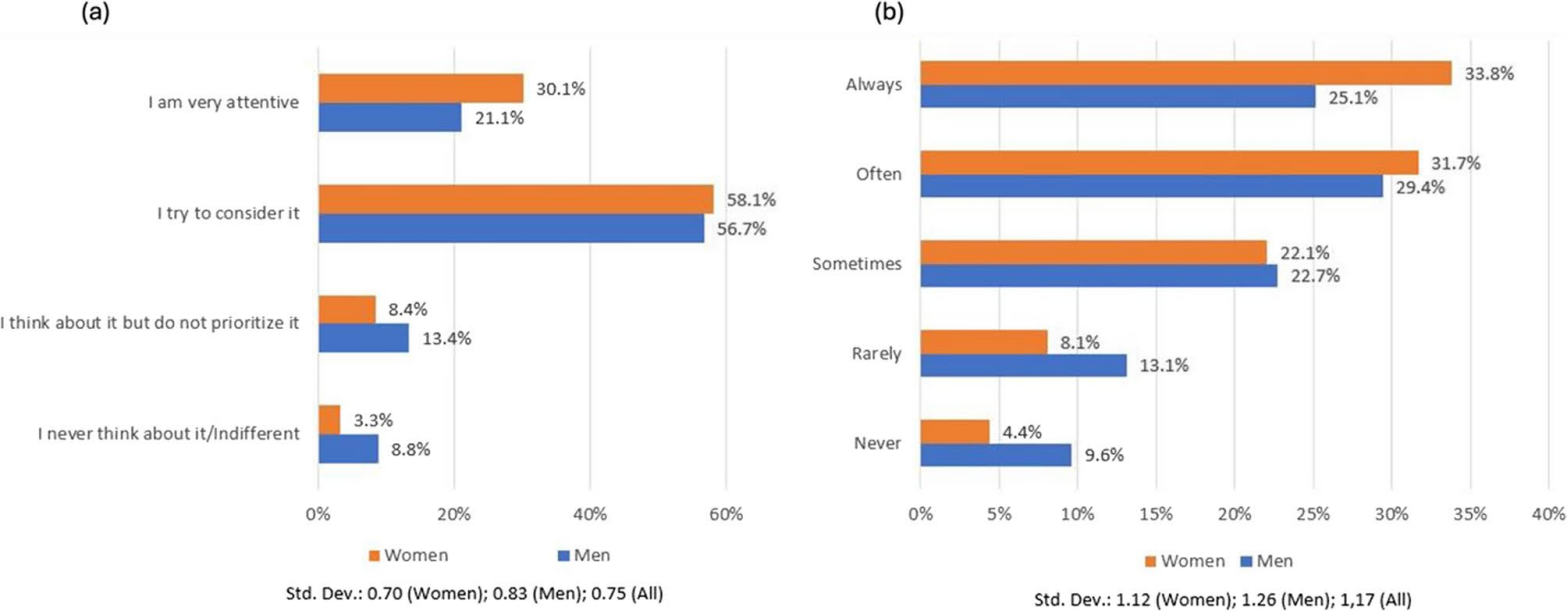
Distribution of QA (How important is animal welfare to you?) and QB (When buying eggs, do you consider how the laying hens were raised?) by gender (QA is shown in panel (**a**); QB is shown in panel (**b**))

A distribution of the answers by gender is shown in Fig. 1 for the two main questions, the former regarding the general animal welfare, the latter going into details on hen welfare. The obtained results suggest that quite similar percentages of men and women tried to pay attention to animal welfare (Fig. 1, (panel (a)), whereas, when hen welfare was analyzed, it was found that women were more attentive than men, even though more than the 50% of the men often or always paid attention to this aspect (Fig. 1, panel (b)).

**Fig. 2:**
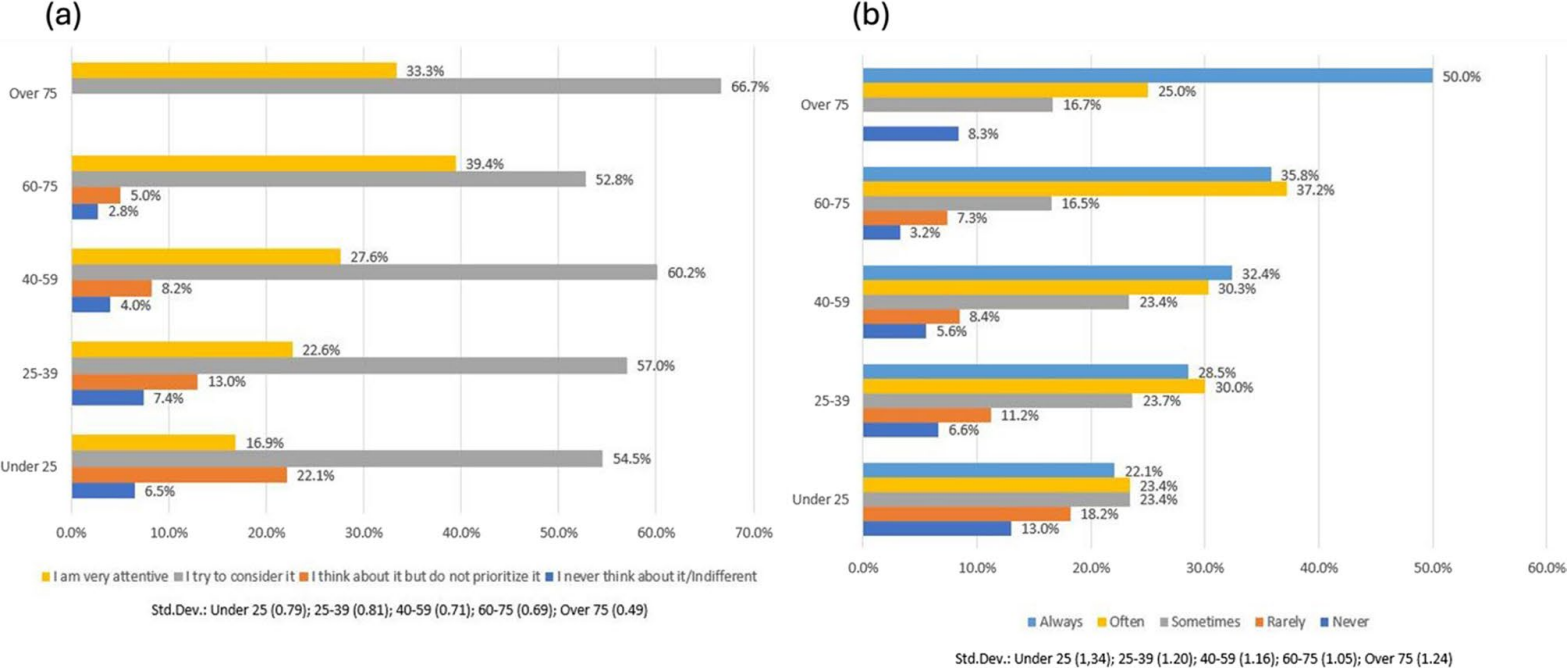
Distribution of QA and QB by age class (QA is shown in panel (**a**); QB is shown in panel (**b**))

Figure 2 describes the distribution of the investigated answers by age class. Most of the respondents, in all the age classes, showed concern about animal welfare (Fig. 2, panel (a)), while a more heterogeneous situation emerged for hen welfare. Indeed, in this second case (Fig. 2, panel (b)), the descriptive statistics showed that consumers over 75 years old were more interested in hen welfare than those below 75 years old, and, in general, it seemed that a positive relationship existed between aging and sensitivity to hen welfare.

The descriptive statistics of the variables introduced into the model and elaborated from the survey are reported in Supplementary Table 1. The regression results are provided in Supplementary Tables 2 and Supplementary Tables 3 and are expressed in terms of odds-ratio. Values higher than 1 describe a positive relationship between the independent variable and the dependent one; values lower than 1 show a negative relationship.

Considering that the difference between model A and B was represented by two dependent variables (“animal welfare’’ in the former and “hen welfare” in the latter), the results suggested that *education and job position* were not statistically significant in either model, while *studying or working in an agri-food sector* increased the attention to animal welfare, although this variable was not statistically significant regarding “hen welfare” (model B). When the number of family members were analyzed, in the “animal welfare” (model A), the coefficients were statistically significant and showed a value lower than 1. This means that the attention to animal welfare decreased as the size of the family increased. The *pet in family* variable was significant in all the models, and the odds- ratio was always higher than 1. The *meat eating* variable was statistically significant in models A and B, with a lower odds-ratio than 1 in model A.4, B.4, B.5, B.6, and B.7. The result of the *welfare-quality* variable was remarkable. Indeed, this variable was statistically significant, showing that participants think that a positive relationship exists between animal welfare and the quality of the product (see “animal welfare” (model A) and “hen welfare” (models B.4, B.5, and B.6)). A similar result was obtained for “hen welfare” (model B), where the variable was not significant in the latter model, although it remained higher than 1. The *color of the eggshell* did not influence the attention of the consumers toward hen welfare, even though, in model B.6, the people who were *willing to buy eggs of different colors* (pink, blue, green, chocolate) were also attentive to hen welfare (positive coefficient, and higher than 1).

As far as the robustness of the results is concerned, complete models A.4 and B.7 showed the highest R-squared values (9.1% and 13.4%, respectively). These were clearly not such high values, and the variance in the results was therefore explained by some factors that were omitted from this analysis. However, the R-squared value allowed us to consider our results convincing. The R-squared statistic quantifies the proportion of variance in the dependent variable that is accounted for by the model. In this context, the results indicate that, on average, the models explain approximately 10% of the variability in the data, a level of explanatory power that is considered acceptable in cross-sectional analyses. Results are particularly robust for hens’ welfare.

The descriptive statistics of the Artificial Intelligence (AI)-based analysis performed considering two questions that were not used in the previous models and adopted in order to analyze their influence on the knowledge of traditional Italian local breeds are presented in Table 1.

**Table 1:** Descriptive statistics on the additional questions for the Stepwise logistic regression analysis. The number of observations (Obs), the mean value (Mean), the standard deviation (Std. Dev.), the minimum and maximum values (Min and Max) are presented in the table for each variable. The respondent had five possible answers to choose from for each option: not at all; A little; quite a lot; A lot; I don’t know

	Variables	Obs	Mean	Std. Dev.	Min	Max
*Advantages of local hen breeds*	*Preserve biodiversity*	1,222	3.741	0.704	1	5
	*Ensure better animal welfare*	1,222	3.681	0.815	1	5
	*Support short supply chains (km 0)*	1,222	3.780	0.671	1	5
*Additional information*	*Laying date*	1,222	0.440	0.497	0	1
	*Breed name (e.g.*,* Bianca di Saluzzo*,* Bionda Piemontese)*	1,222	0.358	0.479	0	1
	Farm location (local, regional, etc.)	1,222	0.665	0.472	0	1
	*Hen diet*	1,222	0.597	0.491	0	1
	*Animal welfare conditions (open-air farming)*	1,222	0.688	0.463	0	1
	*Quality label (e.g.*,* ‘Label Rouge’ in France)*	1,222	0.227	0.419	0	1
	*Environmental sustainability information (e.g.*,* short supply chain*,* circular economy)*	1,222	0.471	0.499	0	1

We adopted a stepwise logistic regression for this analysis, with a significance level of 0.01. The obtained results are presented in Table 2.

**Table 2:** Results of the Stepwise logistic regression analysis – odds ratio. The dependent variable refers to the knowledge of consumers about traditional local Italian breeds (i.e., trad. Local IT breed knowledge). The results are expressed in terms of odds-ratio: >1 = increasing relationship and < 1 = negative relationship

Variables	(C)
	odds ratio
Market (where eggs are bought)	2.516***
	(0.804)
Age (25–39)	0.640***
	(0.109)
Animal welfare	1.691***
	(0.173)
Student (occupation)	0.203***
	(0.069)
Supporting short supply chains (advantage of local breeds)	0.653***
	(0.087)
Own farm (where eggs are bought)	2.129***
	(0.525)
Laying date (additional information)	1.581***
	(0.262)
Breed name (additional information)	1.812***
	(0.320)
Welfare-quality	3.797***
	(0.795)
Employed (occupation)	0.501***
	(0.134)
Constant	2.025
	(1.267)
Observations	1,217
Pseudo R-squared	0.146

Robust seeform in parentheses

*** *p* < 0.01

**Fig. 3:**
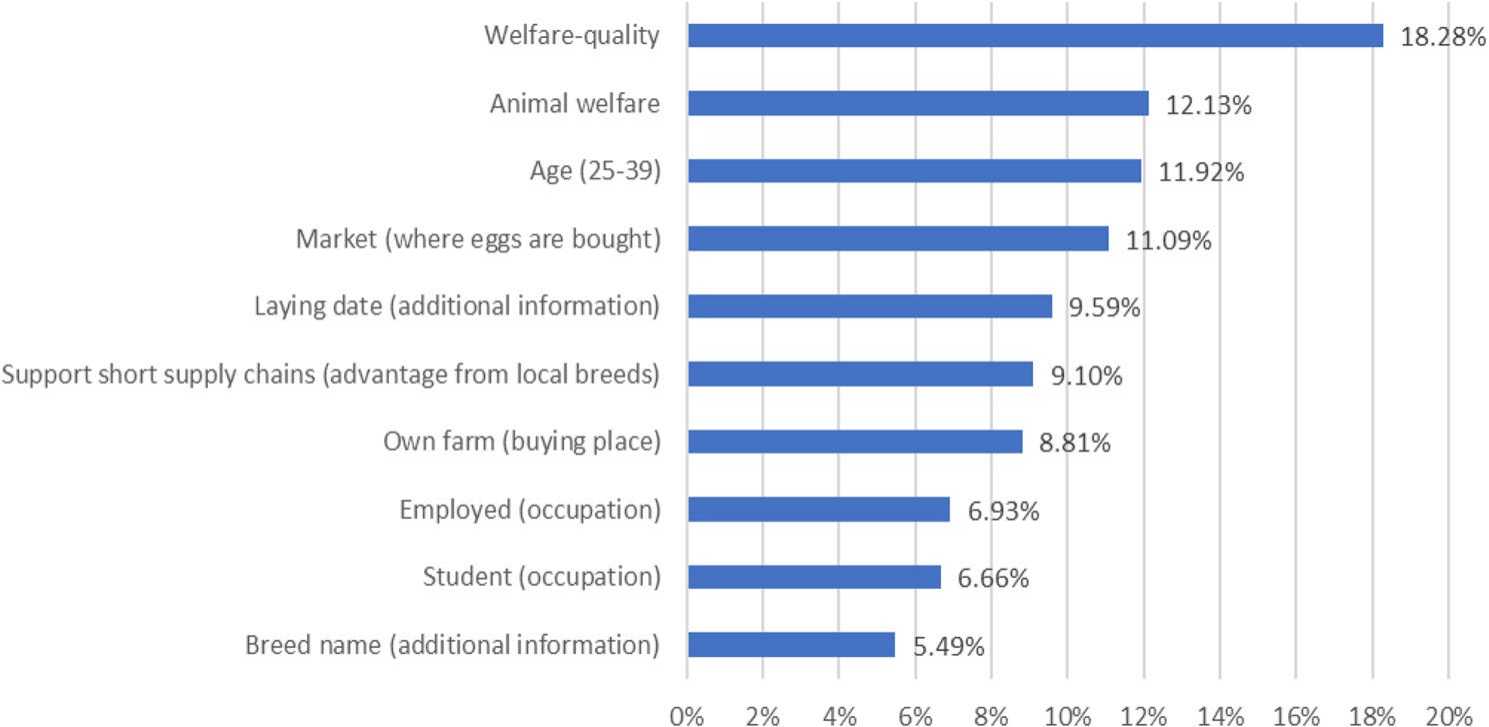
Garson indexes. Quantification of the combined contribution of all input variables by incorporating their interrelationships, as derived from the artificial neural network’s weight structure. Each bar in the histogram represents the contribution of a variable to describing the respondents’ characteristics pertaining to the knowledge of traditional Italian LHB

Figure 3 presents the results of the Garson Indexes obtained with the AI methodology (i.e., Feed-forward Artificial Neural Networks), where the variables are sorted in decreasing order of relevance. Regression analysis estimated the marginal effect of each predictor while holding others constant, whereas Garson’s algorithm, based on artificial neural network weight matrices, assessed the joint influence of all input variables, capturing their interdependencies. In this way, it was possible to compare the contribution of each respondent’s characteristics (i.e., regressors or input-variables), and to define which ones were the most relevant for individuals who had knowledge of Italian hen breeds. According to the findings shown in Fig. 3, the respondents who were familiar with Italian hen breeds were more likely to believe that a relationship existed between the *welfare* of the hens and the *quality* of their eggs. Additional information on *laying date* and *breed name* weighted together for 15% for individuals who knew something about Italian hen breeds. Moreover, these respondents, who were relatively young (25–39 years old) and generally students (18%), demonstrated a high interest in *animal welfare* (12%) and a high awareness of the fact that local breeds could support *short supply chains* (9%).

## Discussion

Although the proposed questionnaire was intended to investigate such a little-known topic as the knowledge of LHBs and their welfare, it nevertheless yielded interesting results. The young population (less than 35 years old) and students showed to be informed on the knowledge of LHBs, while women showed greater concern for hen welfare. Our findings confirm the findings of other studies; for example, it was found that female Mexican consumers exhibited more pro-hen welfare attitudes than men [31]. In an Italian study conducted in 2011, the authors noted that consumers lacked clear information on hen welfare standards and were confused by the current egg labeling. At that time, only 13% of the consumers showed a high interest in knowledge about animal welfare, a higher propensity to purchase animal welfare-friendly products, and a willingness to pay a premium for eggs from alternative systems [32]. However, the situation has significantly improved over the last 12 years, as reported by Harju and colleagues [18]. Indeed, in their survey, over 50% of the respondents attributed a great deal of importance to hen welfare and production methods in their egg purchasing decisions. The situation is still improving, our data showed that more than 60% of the survey’s respondents paid attention to hen welfare.

The age distribution showed that most respondents were concerned about animal welfare; however, older participants were more attentive to the welfare of hens. This result was in contrast with the trends observed in other countries (e.g. China, Brazil, and Europe), where younger generations tended to show greater interest in animal welfare then older ones [18, 33, 34].

Recent investigations have shown that individuals who study or work in the agri-food sector are attentive to animal welfare. For example, researchers found that agronomy students in Costa Rica recognized the importance of animal welfare in livestock production systems and viewed actions that caused unjustified suffering as morally unacceptable [35]. The present study has revealed a similar result for animal welfare, but the results regarding hen welfare have been less convincing. Research conducted in other European and North American countries has highlighted a big empathy and concern for the welfare of dairy cattle, pigs, and horses, which are often perceived as animals that are “close” to humans or as animals that have a greater economic and symbolic value than other animals. Laying hens have instead frequently been marginalized in animal welfare discussions, despite being among the most widely farmed animals in the food industry [36].

According to Tsakiridou and colleagues, family size was one of the main factors that affected consumers’ intention of purchasing welfare-certified animal products [37]. Their findings indicated that a large family size may lead to greater price sensitivity, which in turn could reduce the likelihood of choosing ethically produced products.

Our findings pertaining to people who own pets showing more positive attitudes toward animals in general and, in particular, toward the welfare of livestock on farms, confirm the results of several studies [38–40].

Regarding eating habits, our results showed that *meat-eating* respondents paid less attention to animal/hen welfare. A recent investigation has demonstrated that animal rights and their welfare are almost the strongest motivations for adhering to the dietary goals of vegans and vegetarians, as Vestergren and Uysal reported in a comprehensive review [41]. Although vegans and vegetarians are both non-meat eaters, they are distinct identities. Moral and ethical reasons, which include animal welfare, are more relevant for vegans (80.5%) than for vegetarians (46.7%). In fact, radical animal rights activists are often vegans [42]. A notable finding emerged for the *welfare-product quality* variable. Numerous studies have investigated whether alternative farming systems positively impact animal welfare, and these investigations have shown, contrary to the consumers’ perceptions, that such systems have little or no impact on egg product quality [1, 43]. Nonetheless, the assessment of the effects of alternative housing systems on egg quality is still not conclusive. When such variables as production, mortality, and shell quality were considered, the conventional system presented more convincing results. However, alternative systems (organic free-range and free-range) showed a slightly better nutritional egg quality, despite posing greater management challenges [44].

Our study showed that the individuals who consumed eggs tended to be less attentive to hen welfare than those who did not consume eggs. It is likely that the latter group included vegans, whose dietary choices are strongly motivated by concerns about animal and hen welfare. Furthermore, the price of eggs did not significantly affect the attention of the respondents toward hen welfare. Estévez-Moreno et al. reported similar findings and demonstrated that Mexican consumers showed a high level of concern about the welfare of laying hens, regardless of the egg prices [31].

The relationship between egg color and concern about hen welfare has never been investigated before now. Our study demonstrated that the color of the eggshell did not influence the respondents’ attention to hen welfare, although the individuals who were willing to buy eggs in unconventional colors (pink, blue, green, chocolate) tended to be more attentive to hen welfare. Preferences for eggshell color are influenced by cultural and traditional factors, and they vary significantly—from an almost 100% preference for white shells to 100% for brown shells [45]. White-shell eggs are preferred in Japan, North and Central America, the Middle East, India, Taiwan, and the Philippines, whereas brown-shell eggs are more popular in Latin America, Europe, and China [46].

An interesting finding concerns the respondents who consumed eggs from barn systems (code 2) and who bought unmarked eggs from local farms, as they did not appear to be particularly interested in hen welfare. These were probably people who lived in small rural towns, who bought eggs directly from neighbors, and they showed no feelings about hens being raised intensively. On the contrary, the most attentive consumers to hen welfare were those with knowledge of LHB and those who expressed a willingness to pay more for higher egg quality. Although several studies have explored the willingness to pay for organic eggs and other enhanced production methods (e.g., barn, free-range vs. conventional) [47–49], there are currently no data available on the willingness to pay for eggs produced under an LHB system. In recent years, LHBs have been rediscovered and increasingly appreciated by producers because they are already adapted to the local environment, and consumers’ awareness of these breeds is gradually increasing [50].

The use of innovative analytical tools, such as the stepwise logistic regression method and a shallow feed-forward artificial neural network, allowed us to establish the characteristics of the individuals with knowledge of Italian LHB and how such characteristics weighted on the knowledge of Italian hen breeds. This hybrid approach leveraged on the strengths of both the econometric methodology and artificial intelligence. On one hand, the logistic regression, with its stepwise procedure, allowed more statistically significant variables to be selected, but, on the other hand, the AI-based approach allowed us to order them while considering them all together, and suggested which characteristics were the most important in determining the knowledge of Italian hen breeds. The respondents with knowledge of LHB were mainly part of the younger generation, that is, mainly students, who were informed about environmental issues, and who believed that local breeds could support the short supply chains and produce egg with a high nutritional quality value. The main feature they considered important was related to the improved quality of LHB eggs. Although the evaluation of egg quality from LHB is still ongoing, certain preliminary results, such as those reported by Sirri et al., have indicated promising findings [50]. For instance, eggs produced by the Romagnola hen showed several more valuable egg quality traits than conventional eggs (a higher yolk/egg ratio, more carotenoids, a healthier fatty acid profile), thereby encouraging the promotion of LHB, not only for their heritage value but also from the quality point of view.

Interest in short supply chains has been emerging in recent years, due to an increased awareness of environmental issues. The geographical proximity of production plays a significant role in consumers’ willingness to pay a premium [18]. Cappone et al. demonstrated a close correlation between the local origin of LHB eggs and consumers’ preference, and they confirmed that proximity is a key factor in influencing purchasing decisions [28].

The features revealed by the Garson Indexes could play a key role in planning policies that could be suggested to policymakers to enhance the awareness of LHB among consumers. For example, it is important to acknowledge that dissemination activities, aimed at the young in general and students in particular, are more likely to spread knowledge about LHB, or that indication of the breed of origin and laying date of the eggs would help to spread information about the existence of LHB. There is a clear need to raise awareness about animal and hen welfare through targeted information campaigns. In addition, promoting 0-km products, by emphasizing their freshness and lower energy consumption, could help to preserve the biodiversity of breeds and protect valuable genetic resources that could otherwise be lost.

### Limitations of the study

This study presents some limitations that may influence the possible generalization of the findings. The questionnaire was distributed exclusively via social media, which may have limited participation among the elderly due to potential technological barriers. Moreover, the survey was conducted in Italian, representing a linguistic limitation for foreign consumers. Although it was initially disseminated in the Piedmont region, it expected that it primarily reached respondents in Northern Italy; however, the exact geographical distribution of the responses could not be determined. Other limitations are represented by the characteristics of the LHBs. One of the main challenges is their relatively low egg production, averaging around 150 eggs per hen per year, which is significantly lower than that of commercial laying hens (approximately 300 eggs per year). Moreover, the laying performance of LHBs is strongly influenced by seasonality, showing the maximum peak of egg production in spring and summer. Additionally, LHBs exhibit slower growth rates compared to commercial hybrids, but they are generally better suited to outdoor or extensive farming systems.

## Conclusion

This approach proved highly effective in both identifying and prioritizing the characteristics that defined the consumers’ awareness of local hen breeds. The novelty of this study lies in being the first to investigate such knowledge with a specific focus on hen welfare. The survey revealed that the respondents perceived a direct link between animal and hen welfare and egg quality. Although this relationship has only been marginally investigated so far, it has emerged that it significantly influenced the consumers’ knowledge of hen breeds. People with knowledge of local hen breeds are typically young, educated, and environmentally aware consumers. The ability of policymakers to recognize and respond to this powerful signal from this segment of the population could address the obvious need to shift current farming and breeding paradigms in response to major climate and economic changes. The promotion of short supply chains and egg production based on local hen breeds offers a dual benefit: strengthening consumer confidence and advancing environmental sustainability while safeguarding the genetic heritage of traditional hen breeds. However, it should be noted that LHBs are less productive than commercial lines, which could be a drawback for farmers who are considering adopting this type of breeding system.

In conclusion, outreach about LHBs is needed through social media and popular conferences, supported by dedicated projects, for example on the molecular composition of LHB eggs, that have a spillover effect on the population.

## Methods

### Survey design

The survey was designed to separately analyze the attitude of Italian consumers toward animal welfare and hen welfare, including their knowledge about LHB.

The survey was conducted using the Qualtrics^XM^ online survey software (https://www.qualtrics.com/strategy/research/survey-software/). A draft version of the survey was pre-tested on the authors of this article and their relatives (around 50 participants) to improve the organization and clarify any ambiguity.

The survey consisted of 24 questions, which served to collect demographic data concerning some individual characteristics (i.e., age; gender; education; job position; family characteristics); eating habits (i.e., meat consumption; characteristics of eaten food); purchasing habits and sensitivity to price changes, sensitivity to certain egg characteristics, knowledge about hens and eggs, and finally, attention to animal and hen welfare. The survey was designed in such a way that the participants had to answer all the questions so that no answers were missing for statistical reasons.

An English translation of the administered survey (which had been submitted in Italian) is provided in the Supplementary information section (Supplementary File 1_Survey).

### Survey protocol

An analysis of a sample of interviewed consumers was proposed through a CAWI approach. The interview was administered via social media (Facebook, Instagram, WhatsApp, etc.) from December 6, 2024, till January 7, 2025. A hyperlink was present on each social platform to connect the participants to the survey questions. After an initial dissemination of the survey among selected individuals in the Piedmont region, the authors asked the initial participants to disseminate the survey, and this resulted in a random distribution of the participants. This choice allowed a large number of participants to be collected in a very short time. The survey was developed using a Likert-scale framework, which is a valuable research tool able to capture human sentiments in a standardized manner, enabling respondents to express varying degrees of opinion rather than binary choices [51]. In our case, where applicable, the standard five-point Likert scale was employed, with response options ranging from “Never” (1) to “Always” (5), including “Rarely” (2), “Sometimes” (3), and “Often” (4).

### Definition of the variables and of the ordered logistic model

The following variables were introduced into the model as explanatory items to represent the survey questions (the names of the variables are in italics in the text, and both abbreviated and italicized in the following list):

- *Gen*: this dummy variable was equal to 1 if the respondent was female; 0 if male;
- *Age*: this variable was subdivided into 5 dummy variables to represent 5 different age classes;
- *Edu*: this variable considered 4 levels of education, each represented by a dummy variable;
- *JPos*: was represented by four dummy variables, each representing a different employment status;
- *AFS*: this dummy variable had a value equal to 1 if the respondent worked or studied in an agri-food sector;
- *Fam*: this continuous variable was introduced to represented the number of family members;
- *PF*: this dummy variable was equal to 1 if the respondent had a pet in the house;
- *Mun*: was represented by 5 dummy variables, each representing the size of the municipality where the respondents lived;
- *EM*: this variable was a dichotomous variable equal to 1 if the respondent ate meat, 0 otherwise;
- *WQ*: this dummy variable was equal to 1 if the respondent believed that animal welfare affected the quality of the product;
- *EC* was used to measure the egg consumption habits, and it was represented by 5 dichotomous variables;
- *CC* this variable represented whether the consumer’s consumption of eggs had changed in recent years. Three dummy variables were used to describe increasing/decreasing/unchanged habits;
- *PI*: this dummy variable was equal to 1 if the price of eggs influenced the purchasing decision, 0 otherwise;
- *PB*: was used to indicate the place where a respondent bought eggs. This information was represented by 7 dummy variables;
- *CE*: this dummy variable had a value equal to 1 if the color of the egg affected the consumer’s decision to buy;
- *WC*: this variable took into consideration the color of the egg and it was represented by 3 dummy variables;
- *DC*: this dichotomous variable was equal to 1 if a respondent was willing to buy eggs of a different color, and was equal to 0 otherwise;
- *ET*: this variable was used to consider the type of eggs that a respondent bought. It was represented by 6 dummy variables;
- *Know*: this variable was included to catch whether a respondent knew that there were traditional Italian local breeds that were raised in open spaces. It was a dummy variable that was equal to 1 if the respondent knew;
- *PW*: this dummy variable measured whether a respondent was willing to pay more for a carton of eggs from local Italian hen breeds raised outdoors than for eggs from commercial laying hens raised outdoors, and it was a count variable: an increase in the value indicated an increase is the willingness of a respondent to pay more.

We adopted an ordered logistic regression model, because of the ordinal outcomes of QA and QB (i.e., ‘’animal welfare’’ and ‘’hen welfare’’), with the aim of detecting the characteristics of the consumers who were attentive to animal and hen welfare. The rationale for including two distinct questions lies in the assumption that individuals concerned with animal welfare may not necessarily be attentive to the specific well-being of laying hens.

The respondents were asked to respond to the following two questions: QA. How important is animal welfare to you? The following answers were possible: I never think about it; I think about it but do not prioritize it; I am indifferent to it; I try to consider it; I am very attentive to it. We ordered the answers and merged the “I never think about it” responses with the “I am indifferent to it” ones. QB. When buying eggs, do you consider how the laying hens were raised? The following were possible alternative answers: Never; Rarely; Sometimes; Often; Always.

However, we adopted different models to analyze animal and hen welfare, because the questionnaire was focused on the wellbeing of hens. For this reason, the first three models (A.1; A.2; A.3; B.1; B.2; B.3) were equal, and they considered the same variables. The fourth model (A.4) was the most complete formalization of the models in which animal welfare was the dependent variable (YA). Thus, we formulated another four models (B.4; B.5; B.6; B.7) from the latter, focusing on hen welfare (YB). Results of the models are presented in terms of odds-ratio, and coefficients higher than 1 represented a positive effect of the dependent variable on the independent one; on the contrary, coefficients lower than 1 indicated a decreasing relationship of the regressors on animal or hen welfare. Model C was used to represent a stepwise logistic regression (*p* < 0.01), in which both dependent and independent variables were considered. In the previous model (B.7), the *Trad. IT local breed knowledge (i.e.*,* know)* variable was considered as a regressor, but we also used another methodology, based on Artificial Intelligence (AI), on the two different groups representing the variable. This analysis was designed considering the variables of the previous analysis and another two variables that arose from the questionnaire, and which had not previously been used. The first variable was based on a question that asked the respondents to what extent the production of eggs from LHB could: 1) preserve biodiversity; 2) ensure better animal welfare; 3) support short supply chains (km 0). The respondent could choose from 5 possible answers for each option: Not at all; A little; Quite a lot; A lot; I don’t know. The last variable concerned additional information the respondent would have liked to see on the packaging/label of eggs from local Italian breeds. The logit model with the stepwise estimation, which had a significance level of 0.01, was an iterative procedure, and it allowed us to remove regressors with higher *p*-values than 0.01 from the model [52, 53].

The equations representing the specifications of all the models are reported in Supplementary File 3_Equation Models. STATA 16 software was used for the econometric analysis.

### Artificial neural networks and feature selection

**Fig. 4:**
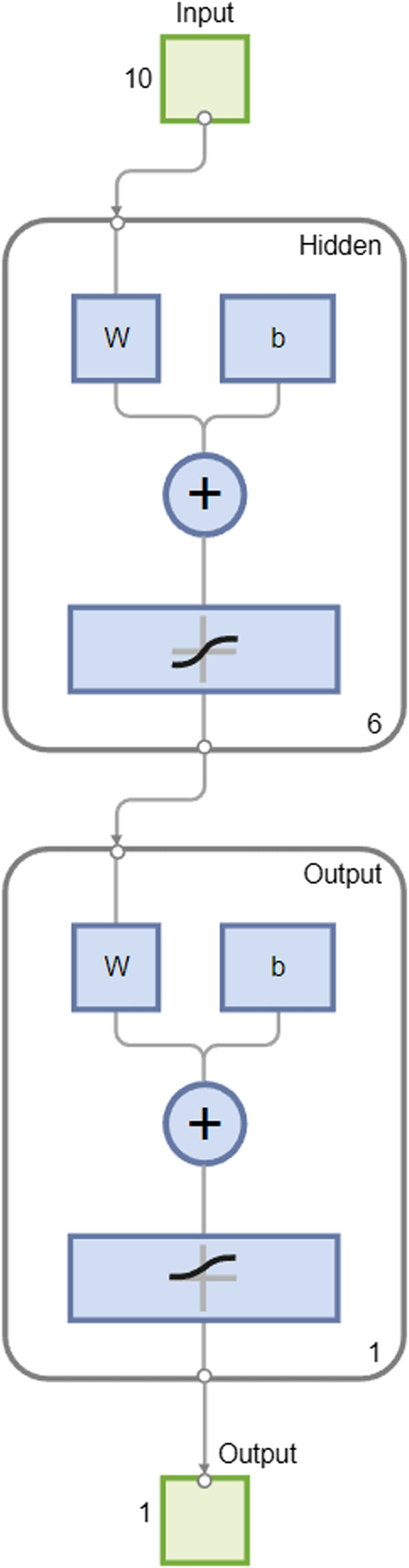
Diagram of the Artificial Neural Network. Note: W: weights, b bias

A deep learning methodology was applied in this work with the aim of classifying the relevance of the various features. A shallow, feed-forward, artificial neural network with two-layers was applied. The first layer called the input layer, consisted of the variables that were introduced into the analysis. The hidden layer was represented by 6 neurons (i.e., the mean between the input and output neurons), and the output layer was represented by only one neuron. Figure 4 presents a diagram of the network.

The real advantage of an artificial neural network is the possibility of introducing more non-linearity relations and of catching any relationships among the data. We applied a tan-sigmoidal function that ranged from − 1 to + 1 between the input and the hidden layer, and a log-sigmoidal function that ranged from − 1 to + 1 between the hidden layer and the output one. The output was represented by the question on the knowledge of Italian hen breeds, and it was aimed at identifying the most significant features.

In addition, according to Calabrese and colleagues [54], we applied conjugate gradient backpropagation with the Fletcher-Reeves algorithm to update the weights [55]. However, we also computed Garson indexes, starting from the weight matrixes that resulted from the layers of the network, to understand the contribution of each variable to the output [56]. Indeed, Garson indexes suggest the contribution of each variable (i.e., feature) to the determination of the output, which, in our case, was the consumer’s knowledge about Italian hen breeds. The Fig. 4 shows the framework of the Artificial Neural Network, which is represented by two layers. The inputs are represented by the ten variables (i.e., ten neurons) that were considered significant by the stepwise logistic regression; the hidden layer is formed by 6 neurons, which are linked to the input neurons through weights (W), bias (b), and a logistic transfer function. The last layer (i.e., the output layer) is represented by one neuron (i.e., knowledge about hen breeds, or not), and it is linked to the hidden layer through weights (W), bias (b), and a tan-sigmoidal transfer function. Literature suggests using the rule of thumb when calculating the mean between the number of neurons in the input and those in the output layers for the computation of the number of neurons in the hidden layer [54]. The Artificial Neural Network and the Garson indexes were computed by using MatlabR2024a software.

## Supplementary Information

Supplementary Material 1: Supplementary File 1_Survey.pdf. Questionnaire: survey on conscious egg consumption. In this file the questionnaire used during the survey is reported, translated in English language.

Supplementary Material 2: Supplementary File 2_Data Collection.xls. Data collected from consumers’ answers.

Supplementary Material 3: Supplementary File 3_Equation Models.pdf. Equations representing the specifications of all the models.

Supplementary Material 4: Supplementary Table 1. Descriptive statistics on the independent and dependent variables.

Supplementary Material 5: Supplementary Table 2.pdf. Ordered logistic regression results on animal welfare.

Supplementary Material 6: Supplementary Table 3.pdf. Ordered logistic regression results on hen welfare.

## Data Availability

Data is provided within the manuscript or supplementary information files.

## Abbreviations

LHB: Local Hen Breeds
CAWI: Computer Assisted Web Interviewing
AI: Artificial Intelligence
