# Supplementary material for: Rediscovering local breeds of naturally free-range hens: a survey on Italian consumers’ awareness of hen welfare and egg purchasing behavior
Source: BMC Vet Res. 2025 Oct 21;21:619. doi: 10.1186/s12917-025-04971-x (PMC12538763; doi:10.1186/s12917-025-04971-x)
Supplement: Supplementary file 1 — Supplementary Material 1: Supplementary File 1_Survey.pdf. Questionnaire: survey on conscious egg consumption. In this file the questionnaire used during the survey is reported, translated in English language. [file 12917_2025_4971_MOESM1_ESM.pdf]

STUDY ON CONSCIOUS EGG CONSUMPTION

SURVEY ON CONSCIOUS EGG CONSUMPTION

Dear participant, we are conducting a study to investigate consumers' preferences regarding egg consumption. This study is part of a research activity on local Italian hen breeds conducted by Prof. Achille Schiavone (UNITO). The questionnaire results from a collaboration with the Institute of Food Production Sciences and the Institute of Research on Sustainable Economic Growth (CNR-IRCrES, https://www.ircres.cnr.it/), both of which are part of the National Research Council (CNR). Your opinion is valuable to help us achieve the research objectives: it will take less than 5 minutes! Thank you for your cooperation.

Participation in the questionnaire is voluntary. The legal basis for data processing is the consent of the participant. All data processing will be carried out while ensuring the anonymity of the individual respondents through data aggregation. Personal data will be processed exclusively for scientific research purposes. By answering the questionnaire, I declare that I have read the privacy policy provided under Article 13 of Legislative Decree 196/2003 and Articles 13 and 14 of EU Regulation 2016/679, and I consent to the processing of the data provided through completion of the questionnaire.
The Privacy Policy information, under Article 13 and subsequent articles of EU Regulation 2016/679, is available at: https://eur-lex.europa.eu/legal-content/IT/TXT/HTML/?uri=CELEX:32016R0679

- I agree

1. Age
   - Under 25
   - 25-39
   - 40-59
   - 60-75
   - Over 75

2. Gender
- Female
- Male
- Non-binary
- Prefer not to disclose

3. What is the highest level of education or training you have successfully completed?
- Elementary school
- Middle school
- Professional school (2-3 years)
- High school (4-5 years)
- Non-university post-qualification training
- Bachelor’s degree
- Master’s degree
- PhD, Specialization, or Postgraduate school

4. Which category best describes your current employment status?
- Employed (employee, employer, self-employed, assistant in family business)
- Unemployed
- Unable to work due to disability or long-term illness
- On parental leave or other leave
- Retired or early retirement
- Full-time homemaker/responsible for household expenses
- Full-time student
- Other (e.g., military service)

5. Do you work or study in the agri-food sector?
- Yes
- No

6. How many members are there in your household?

- 1
- 2
- 3
- 4
- More than 4

7. Do you have a pet in your household?

- Yes
- No

8. Which of the following best describes your living environment?

- Town/city with fewer than 5,000 inhabitants
- Town/city with 5,000-15,000 inhabitants
- Town/city with 15,000-50,000 inhabitants
- Town/city with more than 50,000 inhabitants
- Provincial or regional capital

9. How would you describe your dietary habits? (Choose one option)

- I eat meat, fish, eggs, and dairy products
- I do not eat meat, but I do consume fish, eggs, and dairy products
- I do not eat fish, but I do consume meat, eggs, and dairy products
- I do not eat dairy products, but I do consume meat, fish, and eggs
- I do not eat meat or fish, but I do consume eggs and dairy products
- I do not consume any animal products

10. How often do you consume eggs?

- Never
- At least once a month
- At least twice a month
- Once a week
- Several times a week

11. How has your egg consumption changed in recent years?

- Increased
- Decreased
- Remained unchanged

12. Does the price of eggs influence your purchasing decision?

- Yes
- No
- I don’t know

13. Where do you buy your eggs? (Multiple choices allowed)

- Supermarket
- Traditional grocery store
- Market
- Organic store
- Online shopping/home delivery
- Directly from a farmer
- My own farm

14. Does the color of the eggshell play a role in your decision to buy?
    - Yes
    - No

15. What color of eggshell do you prefer?
    - White
    - Brown
    - No preference

16. What type of eggs do you normally consume? (Multiple choices allowed) (*the eggshell has a numerical code that indicates the kind of husbandry that produces the eggs).
    - Eggs from caged hens (code 3*)
    - Eggs from barn-raised hens (code 2)
    - Eggs from free-range hens (code 1)
    - Eggs from organic farm-raised hens (code 0)
    - Unmarked eggs from local farms
    - No preference/Don't know

17. How important is animal welfare to you?
    - I never think about it
    - I think about it but do not prioritize it
    - I am indifferent about it
    - I try to consider it
    - I am very attentive toward it

18. When buying eggs, do you consider how the laying hens were raised?
    - Never
    - Rarely
    - Sometimes
    - Often
    - Always

19. Do you think animal welfare affects the quality of a product?

- Yes
- No
- I don’t know

20. Are you aware that, besides commercial laying hens (selected for their high productivity in intensive farming systems), there are traditional Italian local breeds that are raised in open spaces?

- Yes
- No

21. Would you be willing to buy eggs of different colors (pink, blue, green, chocolate) from local hen breeders?

- Yes
- No

22. How much do you think the production of local laying hen breeds could help...

	Not at all	A little	Quite a lot	A lot	I don't know
Preserve biodiversity	○	○	○	○	○
Ensure better animal welfare	○	○	○	○	○
Support short supply chains (km 0)	○	○	○	○	○

23. Besides the usual labeling information, what other details would you like to see on the packaging/label of eggs from local Italian breeds? (Multiple answers possible)

- ☐ Laying date
- ☐ Breed name (e.g., Bianca di Saluzzo, Bionda Piemontese)
- ☐ Farm location (local, regional, etc.)
- ☐ Diet of the hens
- ☐ Animal welfare conditions (open-air farming)
- ☐ Quality label (e.g., ‘Label Rouge’ in France)
- ☐ Environmental sustainability information (e.g., short supply chain, circular economy)

24. Would you be willing to pay more for a carton of eggs from local Italian breeds raised outdoors than from commercial laying hens raised outdoors?

- Yes, up to 10% more
- Yes, up to 20% more
- No, I am not willing to pay more
