# Supplementary material for: Rediscovering local breeds of naturally free-range hens: a survey on Italian consumers’ awareness of hen welfare and egg purchasing behavior
Source: BMC Vet Res. 2025 Oct 21;21:619. doi: 10.1186/s12917-025-04971-x (PMC12538763; doi:10.1186/s12917-025-04971-x)
Supplement: Supplementary file 3 — Supplementary Material 3: Supplementary File 3_Equation Models.pdf. Equations representing the specifications of all the models. [file 12917_2025_4971_MOESM3_ESM.pdf]

Equation Models: equations represent the specifications of all the models:

$$Y_i^{A,B} = \beta_0 + \beta_g Gen_i + \beta_a \sum_{a=1}^{A} Age_{a_i} + \beta_e \sum_{e=1}^{E} Edu_{e_i} + \varepsilon_i \quad \text{A.1, B.1}$$

$$Y_i^{A,B} = \beta_0 + \beta_g Gen_i + \beta_a \sum_{a=1}^{A} Age_{a_i} + \beta_e \sum_{e=1}^{E} Edu_{e_i} + \beta_j \sum_{j=1}^{J} JPos_{j_i} + \beta_s AFS_i + \varepsilon_i \quad \text{A.2, B.2}$$

$$Y_i^{A,B} = \beta_0 + \beta_g Gen_i + \beta_a \sum_{a=1}^{A} Age_{a_i} + \beta_e \sum_{e=1}^{E} Edu_{e_i} + \beta_j \sum_{j=1}^{J} JPos_{j_i} + \beta_s AFS_i + \beta_f Fam_i + \beta_p PF_i + \beta_m \sum_{m=1}^{M} Mun_{m_i} + \varepsilon_i \quad \text{A.3, B.3}$$

$$Y_i^{A} = \beta_0 + \beta_g Gen_i + \beta_a \sum_{a=1}^{A} Age_{a_i} + \beta_e \sum_{e=1}^{E} Edu_{e_i} + \beta_j \sum_{j=1}^{J} JPos_{j_i} + \beta_s AFS_i + \beta_f Fam_i + \beta_p PF_i + \beta_m \sum_{m=1}^{M} Mun_{m_i} + \beta_{em} EM_i + \beta_{wq} WQ_i + \varepsilon_i \quad \text{A.4}$$

$$Y_i^{B} = \beta_0 + \beta_g Gen_i + \beta_a \sum_{a=1}^{A} Age_{a_i} + \beta_e \sum_{e=1}^{E} Edu_{e_i} + \beta_j \sum_{j=1}^{J} JPos_{j_i} + \beta_s AFS_i + \beta_f Fam_i + \beta_p PF_i + \beta_m \sum_{m=1}^{M} Mun_{m_i} + \beta_{em} EM_i + \beta_{wq} WQ_i + \beta_{ec} \sum_{ec=1}^{EC} EC_{ec_i} + \beta_{cc} \sum_{cc=1}^{CC} CC_{cc_i} + \varepsilon_i \quad \text{B.4}$$

$$Y_i^{B} = \beta_0 + \beta_g Gen_i + \beta_a \sum_{a=1}^{A} Age_{a_i} + \beta_e \sum_{e=1}^{E} Edu_{e_i} + \beta_j \sum_{j=1}^{J} JPos_{j_i} + \beta_s AFS_i + \beta_f Fam_i + \beta_p PF_i + \beta_m \sum_{m=1}^{M} Mun_{m_i} + \beta_{em} EM_i + \beta_{wq} WQ_i + \beta_{ec} \sum_{ec=1}^{EC} EC_{ec_i} + \beta_{cc} \sum_{cc=1}^{CC} CC_{cc_i} + \beta_{pi} PI_i + \beta_{pb} \sum_{pb=1}^{pb} PB_{pb_i} + \varepsilon_i \quad \text{B.5}$$

$$
\begin{aligned}
{Y_i}^B = \ & \beta_0 + \beta_g Gen_i + \beta_a \sum_{\mathrm{a=1}}^{A} Age_{a_i} + \beta_e \sum_{\mathrm{e=1}}^{E} Edu_{e_i} + \beta_j \sum_{\mathrm{j=1}}^{J} JPos_{j_i} + \beta_s AFS_i \\
& + \beta_f Fam_i + \beta_p PF_i \\
& + \beta_m \sum_{\mathrm{m=1}}^{M} Mun_{m_i} + \beta_{em} EM_i + \beta_{wq} WQ_i + \beta_{ec} \sum_{\mathrm{ec=1}}^{EC} EC_{ec_i} \\
& + \beta_{cc} \sum_{\mathrm{cc=1}}^{CC} CC_{cc_i} + \beta_{pi} PI_i + \beta_{pb} \sum_{\mathrm{pb=1}}^{pb} PB_{pb_i} + \beta_{ce} CE_i \\
& + \beta_{wc} \sum_{\mathrm{wc=1}}^{wc} WC_{wc_i} + \beta_{dc} DC_i + \varepsilon_i
\end{aligned}
\tag{B.6}
$$

$$
\begin{aligned}
{Y_i}^B = \ & \beta_0 + \beta_g Gen_i + \beta_a \sum_{\mathrm{a=1}}^{A} Age_{a_i} + \beta_e \sum_{\mathrm{e=1}}^{E} Edu_{e_i} + \beta_j \sum_{\mathrm{j=1}}^{J} JPos_{j_i} + \beta_s AFS_i \\
& + \beta_f Fam_i + \beta_p PF_i \\
& + \beta_m \sum_{\mathrm{m=1}}^{M} Mun_{m_i} + \beta_{em} EM_i + \beta_{wq} WQ_i + \beta_{ec} \sum_{\mathrm{ec=1}}^{EC} EC_{ec_i} \\
& + \beta_{cc} \sum_{\mathrm{cc=1}}^{CC} CC_{cc_i} + \beta_{pi} PI_i + \beta_{pb} \sum_{\mathrm{pb=1}}^{pb} PB_{pb_i} + \beta_{ce} CE_i \\
& + \beta_{wc} \sum_{\mathrm{wc=1}}^{wc} WC_{wc_i} + \beta_{dc} DC_i + \beta_{et} \sum_{\mathrm{et=1}}^{et} ET_{et_i} + \beta_{ki} Know_i + \beta_{pd} PD_i \\
& + \varepsilon_i
\end{aligned}
\tag{B.7}
$$

$$
\begin{aligned}
{Y_i}^C = \ & \beta_0 + \beta_g Gen_i + \beta_a \sum_{\mathrm{a=1}}^{A} Age_{a_i} + \beta_e \sum_{\mathrm{e=1}}^{E} Edu_{e_i} + \beta_j \sum_{\mathrm{j=1}}^{J} JPos_{j_i} + \beta_s AFS_i \\
& + \beta_f Fam_i + \beta_p PF_i \\
& + \beta_m \sum_{\mathrm{m=1}}^{M} Mun_{m_i} + \beta_{em} EM_i + \beta_{wq} WQ_i + \beta_{ec} \sum_{\mathrm{ec=1}}^{EC} EC_{ec_i} \\
& + \beta_{cc} \sum_{\mathrm{cc=1}}^{CC} CC_{cc_i} + \beta_{pi} PI_i + \beta_{pb} \sum_{\mathrm{pb=1}}^{pb} PB_{pb_i} + \beta_{ce} CE_i \\
& + \beta_{wc} \sum_{\mathrm{wc=1}}^{wc} WC_{wc_i} + \beta_{dc} DC_i + \beta_{et} \sum_{\mathrm{et=1}}^{et} ET_{et_i} + \beta_{ki} Know_i + \beta_{pd} PD_i \\
& + \beta_{alb} \sum_{\mathrm{alb=1}}^{ALB} ALB_{alb_i} + \beta_{ai} \sum_{\mathrm{ai=1}}^{AI} AI_{ai_i} + \varepsilon_i
\end{aligned}
\tag{C}
$$
