# Supplementary material for: Rediscovering local breeds of naturally free-range hens: a survey on Italian consumers’ awareness of hen welfare and egg purchasing behavior
Source: BMC Vet Res. 2025 Oct 21;21:619. doi: 10.1186/s12917-025-04971-x (PMC12538763; doi:10.1186/s12917-025-04971-x)
Supplement: Supplementary file 4 — Supplementary Material 4: Supplementary Table 1. Descriptive statistics on the independent and dependent variables. [file 12917_2025_4971_MOESM4_ESM.pdf]

Table 1: Descriptive statistics on the independent and dependent variables. The number of observations (Obs), the mean value (Mean), the standard deviation (Std. Dev.), the minimum and the maximum values (Min and Max) are reported in the table for each variable.

			**Obs**	**Mean**	**Std. Dev.**	**Min**	**Max**
Dependent variables	Y^A	Animal welfare	1,222	3.075	0.754	1	4
	Y^B	Hen welfare	1,222	3.718	1.175	1	5
Independent variables	*Gen*	Gender (dummy variable)	1,217	0.693	0.462	0	1
	Age	*Age classes (dummy variables)*					
		<25	1,222	0.063	0.243	0	1
		25<Age<39	1,222	0.322	0.467	0	1
		40<Age<59	1,222	0.427	0.495	0	1
		60<Age<75	1,222	0.178	0.383	0	1
		75<Age	1,222	0.010	0.099	0	1
	Edu	*Education*					
		Elementary-school education	1,222	0.085	0.279	0	1
		Middle-school	1,222	0.279	0.449	0	1
		High-school education	1,222	0.191	0.393	0	1
		Degree education	1,222	0.267	0.442	0	1
		Further-degree education	1,222	0.178	0.383	0	1
	JPos	*Job position (dummy variables)*					
		Unemployed/Homemaker	1,222	0.053	0.225	0	1
		Employed	1,222	0.726	0.446	0	1
		Retired	1,222	0.123	0.328	0	1
		Student	1,222	0.088	0.284	0	1
		Other	1,222	0.010	0.099	0	1
	AFS	Agri-food sector *(dummy variable)*	1,222	0.190	0.392	0	1
	Fam	Family members (cont.)	1,222	2.862	1.228	1	5
	PF	Pet in family *(dummy variable)*	1,222	0.560	0.497	0	1
	Mun	*Municipality population (dummy variables)*					
		<5,000 inhabitants	1,222	0.230	0.421	0	1
		5,000<inhabitants<15,000	1,222	0.227	0.419	0	1
		15,000<inhabitants<50,000	1,222	0.263	0.440	0	1
		Inhabitants>50,000	1,222	0.054	0.226	0	1
		Provincial/Regional Capital	1,222	0.227	0.419	0	1
	EM	Meat eating *(dummy variable)*	1,222	0.872	0.334	0	1
	WQ	Welfare-quality *(dummy variable)*	1,222	0.883	0.322	0	1
	EC	*Egg consumption (dummy variables)*					
		Never	1,222	0.007	0.086	0	1
		Once a month	1,222	0.064	0.245	0	1
		Twice a month	1,222	0.446	0.497	0	1
		Once a week	1,222	0.177	0.382	0	1
		Several times a week	1,222	0.306	0.461	0	1
	CC	*Change in consumption (dummy variables)*					
		Unchanged	1,222	0.615	0.487	0	1
		Increased consumption	1,222	0.227	0.419	0	1
		Decreased consumption	1,222	0.158	0.365	0	1
	PI	Influenced by price *(dummy variable)*	1,222	0.186	0.389	0	1
	PB	*Place where eggs are bought (dummy variables)*					

	Supermarket	1,222	0.710	0.454	0	1
	Traditional grocery store	1,222	0.151	0.358	0	1
	Market	1,222	0.136	0.343	0	1
	Own farm	1,222	0.145	0.352	0	1
	Organic store	1,222	0.071	0.257	0	1
	Online shopping/home delivery	1,222	0.017	0.130	0	1
	Directly from a farmer	1,222	0.298	0.458	0	1
CE	Effect of the egg color *(dummy variable)*	1,222	0.144	0.351	0	1
WC	*What color? (dummy variables)*					
	None	1,222	0.232	0.423	0	1
	White	1,222	0.101	0.302	0	1
	Brown	1,222	0.666	0.472	0	1
DC	Buying eggs of different colors*(dummy variable)*	1,222	0.842	0.365	0	1
ET	*Type of eggs (dummy variables)*					
	No preference	1,222	0.288	0.453	0	1
	Code 3	1,222	0.012	0.110	0	1
	Code 2	1,222	0.360	0.480	0	1
	Code 1	1,222	0.429	0.495	0	1
	Code 0	1,222	0.411	0.492	0	1
	Unmarked eggs from local farms	1,222	0.110	0.314	0	1
Know	Knowledge of local IT breeds *(dummy variable)*	1,222	0.810	0.392	0	1
PW	Willingness to pay (count)	1,222	1.302	0.635	0	2
