# Supplementary material for: Rediscovering local breeds of naturally free-range hens: a survey on Italian consumers’ awareness of hen welfare and egg purchasing behavior
Source: BMC Vet Res. 2025 Oct 21;21:619. doi: 10.1186/s12917-025-04971-x (PMC12538763; doi:10.1186/s12917-025-04971-x)
Supplement: Supplementary file 5 — Supplementary Material 5: Supplementary Table 2.pdf. Ordered logistic regression results on animal welfare. [file 12917_2025_4971_MOESM5_ESM.pdf]

Table 2: Ordered logistic regression results on animal welfare (odds-ratio). In this model, the dependent variable is represented by animal welfare. The four model specifications progressively consider an increasing number of regressors to explain the relationship between the regressors and the propension to consider animal welfare. The obtained results are expressed in terms of odds-ratio: a value higher than 1 represents an increasing relationship, while values below 1 indicate the relationship is negative.

VARIABLES	(A.1) odds ratio	(A.2) odds ratio	(A.3) odds ratio	(A.4) odds ratio
Gender (1 female)	1.809***	1.872***	1.889***	1.716***
	(0.233)	(0.244)	(0.248)	(0.230)
Age classes				
Benchmark (Age< 25)				
25<Age<39	1.420	1.767**	1.711*	1.575
	(0.370)	(0.501)	(0.503)	(0.474)
40<Age<59	2.041***	2.910***	2.932***	2.891***
	(0.519)	(0.858)	(0.891)	(0.901)
60<Age<75	3.674***	4.163***	4.276***	4.196***
	(1.011)	(1.476)	(1.545)	(1.562)
75<Age	4.120***	4.190***	4.315***	4.908***
	(1.905)	(2.313)	(2.257)	(2.711)
Education				
Benchmark (Elementary-school education)				
Middle-school	0.672*	0.731	0.743	0.726
	(0.162)	(0.179)	(0.183)	(0.184)
High-school education	0.922	1.033	1.015	0.864
	(0.243)	(0.275)	(0.271)	(0.240)
Degree education	0.743	0.806	0.863	0.850
	(0.184)	(0.203)	(0.220)	(0.224)
Further-degree education	0.882	0.950	1.024	0.989
	(0.231)	(0.255)	(0.277)	(0.274)
Job position				
Benchmark (other)				
Unemployed/Homemaker		0.915	0.886	0.836
		(0.630)	(0.634)	(0.566)
Employed		0.714	0.705	0.684
		(0.466)	(0.475)	(0.431)
Retired		1.118	1.028	0.899
		(0.797)	(0.752)	(0.620)
Student		0.920	1.025	0.869
		(0.648)	(0.747)	(0.602)
Agri-food sector		1.724***	1.737***	1.627***
		(0.265)	(0.270)	(0.260)
Family members			0.891**	0.924
			(0.046)	(0.048)

Pet in family			1.844***	1.777***
			(0.224)	(0.219)
Municipality population				
Benchmark (<5,000 inhabitants)				
5,000<inhabitants<15,000			0.810	0.790
			(0.133)	(0.136)
15,000<inhabitants<50,000			0.932	0.874
			(0.154)	(0.152)
Inhabitants>50,000			0.916	0.973
			(0.256)	(0.272)
Provincial/Regional Capital			0.872	0.929
			(0.152)	(0.168)
Meat eating				0.205***
				(0.039)
Welfare-quality				3.093***
				(0.571)
/cut1	0.111***	0.132***	0.119***	0.060***
	(0.038)	(0.100)	(0.094)	(0.048)
/cut2	0.380***	0.459	0.415	0.217*
	(0.128)	(0.345)	(0.324)	(0.172)
/cut3	6.557***	8.190***	7.855***	4.969**
	(2.224)	(6.142)	(6.119)	(3.919)
Observations	1,217	1,217	1,217	1,217
Pseudo R-squared	0.0281	0.0353	0.0475	0.0910

Robust seeform in parentheses
*** p<0.01, ** p<0.05, * p<0.1
