# Supplementary material for: Rediscovering local breeds of naturally free-range hens: a survey on Italian consumers’ awareness of hen welfare and egg purchasing behavior
Source: BMC Vet Res. 2025 Oct 21;21:619. doi: 10.1186/s12917-025-04971-x (PMC12538763; doi:10.1186/s12917-025-04971-x)
Supplement: Supplementary file 6 — Supplementary Material 6: Supplementary Table 3.pdf. Ordered logistic regression results on hen welfare. [file 12917_2025_4971_MOESM6_ESM.pdf]

**Table 3:** Ordered logistic regression results on hen welfare (odds-ratio). The dependent variable is represented by hen welfare. The four model specifications progressively consider an increasing number of regressors to explain the relationship between the regressors and the propension to consider hen welfare. The obtained results are expressed in terms of odds-ratio: value > 1 = increasing relationship; value < 1 negative relationship.

| VARIABLES | (B.1) odds ratio | (B.2) odds ratio | (B.3) odds ratio | (B.4) odds ratio | (B.5) odds ratio | (B.6) odds ratio | (B.7) odds ratio |
|---|---|---|---|---|---|---|---|
| Gender (1 female) | 1.625*** | 1.697*** | 1.712*** | 1.556*** | 1.580*** | 1.571*** | 1.472*** |
| | (0.189) | (0.200) | (0.204) | (0.187) | (0.192) | (0.193) | (0.187) |
| *Age classes* | | | | | | | |
| *Benchmark (Age< 25)* | | | | | | | |
| 25<Age<39 | 1.581* | 2.242*** | 2.236*** | 2.020** | 1.999** | 1.958** | 2.279*** |
| | (0.401) | (0.589) | (0.600) | (0.564) | (0.563) | (0.555) | (0.666) |
| 40<Age<59 | 1.897** | 3.098*** | 3.111*** | 2.927*** | 2.967*** | 2.999*** | 2.737*** |
| | (0.473) | (0.854) | (0.865) | (0.850) | (0.873) | (0.886) | (0.830) |
| 60<Age<75 | 2.863*** | 3.220*** | 3.164*** | 3.003*** | 3.006*** | 3.243*** | 2.510*** |
| | (0.755) | (0.987) | (0.988) | (0.977) | (0.990) | (1.076) | (0.864) |
| 75<Age | 4.577** | 4.048** | 4.010** | 4.265** | 4.445** | 4.818** | 5.584** |
| | (2.887) | (2.778) | (2.655) | (2.927) | (3.124) | (3.372) | (3.955) |
| *Education* | | | | | | | |
| *Benchmark* (Elementary-school education) | | | | | | | |
| Middle-school | 0.937 | 1.034 | 1.040 | 1.061 | 1.157 | 1.078 | 1.042 |
| | (0.193) | (0.214) | (0.217) | (0.223) | (0.250) | (0.236) | (0.239) |
| High-school education | 1.194 | 1.339 | 1.283 | 1.204 | 1.321 | 1.225 | 1.120 |
| | (0.269) | (0.302) | (0.291) | (0.280) | (0.310) | (0.290) | (0.277) |
| Degree education | 1.217 | 1.355 | 1.325 | 1.363 | 1.553* | 1.424 | 1.190 |
| | (0.258) | (0.291) | (0.291) | (0.306) | (0.356) | (0.331) | (0.294) |
| Further-degree education | 1.345 | 1.515* | 1.461 | 1.446 | 1.595* | 1.442 | 1.204 |
| | (0.309) | (0.355) | (0.347) | (0.346) | (0.392) | (0.359) | (0.316) |
| *Job position* | | | | | | | |
| *Benchmark (other)* | | | | | | | |
| Unemployed/Homemaker | | 0.897 | 0.932 | 0.851 | 0.786 | 0.842 | 0.891 |
| | | (0.625) | (0.662) | (0.583) | (0.523) | (0.551) | (0.594) |
| Employed | | 0.726 | 0.737 | 0.731 | 0.565 | 0.605 | 0.753 |
| | | (0.488) | (0.505) | (0.480) | (0.358) | (0.378) | (0.477) |
| Retired | | 1.478 | 1.446 | 1.294 | 0.951 | 0.967 | 1.201 |
| | | (1.039) | (1.038) | (0.894) | (0.640) | (0.643) | (0.814) |
| Student | | 1.289 | 1.432 | 1.297 | 1.082 | 1.153 | 1.492 |
| | | (0.898) | (1.017) | (0.882) | (0.711) | (0.746) | (1.002) |
| Agri-food sector | | 1.466*** | 1.441** | 1.321* | 1.315* | 1.280 | 1.150 |
| | | (0.211) | (0.212) | (0.201) | (0.205) | (0.200) | (0.184) |
| Family members | | | 0.905** | 0.922* | 0.912* | 0.919* | 0.962 |
| | | | (0.043) | (0.045) | (0.045) | (0.045) | (0.046) |
| Pet in family | | | 1.296** | 1.248** | 1.210* | 1.233* | 1.239* |
| | | | (0.142) | (0.138) | (0.136) | (0.140) | (0.142) |

| | | | | | |
|---|---|---|---|---|---|
| *Municipality population* | | | | | |
| *Benchmark (<5,000 inhabitants)* | | | | | |
| 5,000<inhabitants<15,000 | 0.776* | 0.769* | 0.796 | 0.795 | 0.764 |
| | (0.116) | (0.117) | (0.123) | (0.124) | (0.127) |
| 15,000<inhabitants<50,000 | 1.041 | 1.003 | 1.056 | 1.062 | 1.043 |
| | (0.160) | (0.158) | (0.170) | (0.171) | (0.172) |
| Inhabitants>50,000 | 0.959 | 0.981 | 1.175 | 1.195 | 1.279 |
| | (0.295) | (0.299) | (0.356) | (0.367) | (0.376) |
| Provincial/Regional Capital | 1.022 | 1.052 | 1.215 | 1.192 | 1.130 |
| | (0.167) | (0.176) | (0.215) | (0.212) | (0.206) |
| Meat eating | | 0.283*** | 0.284*** | 0.288*** | 0.364*** |
| | | (0.050) | (0.052) | (0.053) | (0.069) |
| Welfare-quality | | 1.785*** | 1.559** | 1.627** | 1.058 |
| | | (0.319) | (0.293) | (0.309) | (0.196) |
| *Egg consumption* | | | | | |
| *Benchmark (Never)* | | | | | |
| Once a month | | 0.217* | 0.285 | 0.292 | 0.157** |
| | | (0.183) | (0.238) | (0.258) | (0.144) |
| Twice a month | | 0.348 | 0.447 | 0.432 | 0.184* |
| | | (0.284) | (0.359) | (0.366) | (0.165) |
| Once a week | | 0.255* | 0.339 | 0.324 | 0.156** |
| | | (0.209) | (0.273) | (0.276) | (0.139) |
| Several times a week | | 0.352 | 0.435 | 0.420 | 0.197* |
| | | (0.289) | (0.351) | (0.357) | (0.176) |
| *Change in consumption* | | | | | |
| *Benchmark (Unchanged)* | | | | | |
| Increased consumption | | 1.128 | 1.104 | 1.099 | 1.060 |
| | | (0.150) | (0.149) | (0.150) | (0.147) |
| Decreased consumption | | 1.097 | 1.127 | 1.141 | 1.218 |
| | | (0.193) | (0.196) | (0.197) | (0.214) |
| Influenced by price | | | 0.689*** | 0.693*** | 0.906 |
| | | | (0.093) | (0.094) | (0.127) |
| *Place of buying eggs* | | | | | |
| *Benchmark (Supermarket)* | | | | | |
| Traditional grocery store | | | 0.973 | 0.948 | 0.934 |
| | | | (0.149) | (0.144) | (0.145) |
| Market | | | 1.197 | 1.176 | 1.074 |
| | | | (0.194) | (0.193) | (0.170) |
| Own farm | | | 2.047*** | 2.033*** | 1.772*** |
| | | | (0.351) | (0.354) | (0.321) |
| Organic store | | | 1.940*** | 1.969*** | 1.121 |
| | | | (0.416) | (0.435) | (0.272) |
| Online shopping/home delivery | | | 0.902 | 0.887 | 0.879 |
| | | | (0.382) | (0.371) | (0.303) |
| Directly from a farmer | | | 1.883*** | 1.852*** | 1.469*** |
| | | | (0.233) | (0.232) | (0.193) |

| | | | | | | | |
|---|---|---|---|---|---|---|---|
| *Effect of the color of the eggshell* | | | | | | 1.117 | 1.177 |
| | | | | | | (0.207) | (0.225) |
| *What color?* | | | | | | | |
| *Benchmark (None)* | | | | | | | |
| White | | | | | | 1.136 | 0.948 |
| | | | | | | (0.232) | (0.192) |
| Brown | | | | | | 1.153 | 1.019 |
| | | | | | | (0.181) | (0.165) |
| *Buying eggs of different colors* | | | | | | 1.588*** | 1.238 |
| | | | | | | (0.264) | (0.218) |
| *Type of eggs* | | | | | | | |
| *Benchmark (No preference)* | | | | | | | |
| Code 3 | | | | | | | 0.533 |
| | | | | | | | (0.286) |
| Code 2 | | | | | | | 0.457*** |
| | | | | | | | (0.060) |
| Code 1 | | | | | | | 1.071 |
| | | | | | | | (0.127) |
| Code 0 | | | | | | | 1.619*** |
| | | | | | | | (0.208) |
| Unmarked eggs from local farms | | | | | | | 0.173*** |
| | | | | | | | (0.039) |
| Trad. IT local breed knowledge | | | | | | | 1.629*** |
| | | | | | | | (0.259) |
| Willingness to pay | | | | | | | 1.961*** |
| | | | | | | | (0.218) |
| /cut1 | 0.178*** | 0.250* | 0.205** | 0.030*** | 0.037*** | 0.063** | 0.034*** |
| | (0.056) | (0.185) | (0.159) | (0.033) | (0.040) | (0.070) | (0.041) |
| /cut2 | 0.526** | 0.747 | 0.612 | 0.092** | 0.115** | 0.195 | 0.119* |
| | (0.161) | (0.557) | (0.479) | (0.100) | (0.123) | (0.216) | (0.143) |
| /cut3 | 1.791* | 2.585 | 2.133 | 0.332 | 0.434 | 0.743 | 0.565 |
| | (0.546) | (1.929) | (1.669) | (0.362) | (0.463) | (0.823) | (0.677) |
| /cut4 | 6.720*** | 9.899*** | 8.276*** | 1.382 | 1.914 | 3.306 | 3.114 |
| | (2.079) | (7.407) | (6.491) | (1.505) | (2.040) | (3.661) | (3.731) |
| Observations | 1,217 | 1,217 | 1,217 | 1,217 | 1,217 | 1,217 | 1,217 |
| Pseudo R-squared | 0.0138 | 0.0208 | 0.0247 | 0.0474 | 0.0641 | 0.0671 | 0.134 |

Robust seeform in parentheses

*** $p<0.01$, ** $p<0.05$, * $p<0.1$
